# Expectations and Preferences for Digital Cessation Treatment: Multimethods Study Among Older Adults Who Smoke Cigarettes

**DOI:** 10.2196/52919

**Published:** 2024-08-28

**Authors:** Margaret C Fahey, Mathew J Carpenter, Riley O'Neal, Kinsey Pebley, Melissa R Schick, Emily Ware, Benjamin A Toll, Jennifer Dahne

**Affiliations:** 1 Department of Psychiatry & Behavioral Sciences Medical University of South Carolina Charleston, SC United States; 2 Hollings Cancer Center Charleston, SC United States; 3 School of Arts & Sciences University of South Carolina Columbia, SC United States; 4 Department of Public Health Sciences Medical University of South Carolina Charleston, SC United States; 5 School of Medicine Yale University New Haven, CT United States; 6 Department of Pharmacy Services Medical University of South Carolina Charleston, SC United States

**Keywords:** older adults, digital health, digital cessation treatment, smoking cessation, digital cessation, treatment, smoke, cigarettes, cigarette, tobacco, adults, elderly, older person, older people, aging, smoking, smoke, quit, quitting, questionnaire, telehealth, treatment, treatments, behavioral health, public health, mobile phone

## Abstract

**Background:**

To address enduring age-related tobacco disparities, it is critical to promote cessation treatment among older adults (aged 65+ years). Digital health platforms offer opportunities for wide dissemination of evidence-based behavioral cessation support. However, existing digital cessation treatments are not tailored to unique aging-related needs and preferences, resulting in low uptake. Detailed information is needed about how to best adapt these treatments for this age group.

**Objective:**

We aimed to collect detailed, hypothesis-generating information about expectations and preferences for cessation digital treatment among older adults who smoke cigarettes.

**Methods:**

Semistructured interviews were conducted with adults aged 65+ years currently smoking or who had quit within the past month. Interviews included open-ended questions regarding prior experiences with digital health platforms and expectations and preferences for cessation treatment via various modalities (app-delivered, texting-based, or videoconferencing counseling). Interviews also elicited questions regarding digital modalities that integrated social components (app-delivered social forums and group videoconferencing counseling). Using an iterative, team-based approach, the thematic analysis identified meaningful themes. Interviews were supplemented with quantitative measures assessing sociodemographics, digital literacy, and physical health symptoms.

**Results:**

Participants (12/20, 60% men; 15/20, 75% White; 4/20, 20% Black or African American; 1/20, 5% Asian) were currently smoking (17/20, 85%) or had recently quit (3/20, 15%). Thematic analysis identified 3 meaningful themes across all digital modalities: convenience, accessibility, and personalization. Expected benefits of digital platforms included convenient treatment access, without reliance on transportation. Participants preferred treatments to be personalized and deliver content or strategies beyond standard education. Most (17/20, 85%) were unfamiliar with cessation apps but found them appealing given the potential for offering a novel quitting strategy. App ease of use (eg, easy navigation) was preferred. Half (10/20, 50%) would try a texting-based intervention, with many preferring texting with a counselor rather than automated messaging. Most (17/20, 85%) would use videoconferencing and expected this modality to deliver better quality counseling than via telephone. Expected videoconferencing challenges included looking presentable onscreen, technological difficulties, and privacy or security. Videoconferencing was regarded as the most personalized digital treatment, yet benefits unique to app-delivered and texting-based treatments included anonymity and access to treatment 24/7. Participants expected integrating social components into digital treatment to be useful for quit success and social connection, yet were concerned about possible interpersonal challenges.

**Conclusions:**

Because a long history of quit attempts and familiarity with standard quitting advice is common among older adults who smoke cigarettes, digital platforms might offer appealing and novel strategies for cessation that are accessible and convenient. Overall, this population was open to trying digital cessation treatments and would prefer that these platforms prioritize ease of use and personalized content. These findings challenge the bias that older adults are uninterested or unwilling to engage with digital treatments for behavioral health.

## Introduction

In total, 1 in 7 Americans is aged 65 years or older, an age group that will comprise 22% of the US population by 2040 [1]. Cigarette smoking is a leading cause of cancer, preventable disease, and death in the United States, with tobacco morbidity and mortality disproportionately impacting older adults [2]. Cigarette smoking exacerbates numerous chronic health conditions that are more common in older age (eg, diabetes or chronic pain) [2-5]. Annual smoking-related mortality is almost twice as high for older (392,000 deaths) compared to younger (163,000 deaths) adults [2]. Although younger age groups have seen significant declines, smoking prevalence has stagnated among older adults (9%) for the past 15 years [6-9]. Despite lower smoking prevalence in older compared to younger adults, this difference is attributed to older adults being much more likely to die from smoking [8,10]. Fortunately, cessation offers significant health advantages in older age, including improved physical health and increased lifespan [2-4,11,12]. To address these age-related tobacco disparities, there is a critical need to promote the use of evidence-based cessation treatments in this age group.

More than half of older adults who smoke cigarettes want to quit [13], but only about a third (37%) use an evidence-based treatment when making a quit attempt [14]. However, when older adults do engage in evidence-based treatment, they experience comparable (and sometimes higher) quit rates compared to their younger counterparts [15]. Digital health treatment modalities offer opportunities for the wide dissemination of evidence-based behavioral cessation support (eg, mobile apps, texting programs, or counseling via videoconferencing) [16]. Digital platforms can overcome in-person barriers (eg, physical limitations or lack of transportation) common in older age, increase the availability of treatment content (eg, at times of cravings), and supplement limited provider time in medical settings [16-18]. Further, ownership of consumer technologies is increasing in this age group, as 88% of US adults aged 56-70 years and 72% aged 70+ years owned smartphones in 2022 compared to 81% and 62%, respectively, in 2021 [19]. Digital treatments effectively promote a variety of other health behaviors (eg, physical activity) in later life [20,21] and could be a means to widely disseminate cessation treatment to this high-priority population.

Despite the promise of digital health for this age group, to our knowledge, no digital cessation treatment is tailored specifically to unique aging-related needs and preferences, despite many focused on adolescent or young adult populations [16,22-24]. The lack of tailored support for this age group might contribute to low uptake. For example, the National Cancer Institute Smokefree.gov app-delivered cessation programs are free and produce quit rates comparable to other behavioral interventions, but only 2% to 3% of its users are aged 65+ years [22,25]. Further, the Washington Department of Health provides a freely available app-delivered program, yet older adults comprise <3% of those using this program (despite comprising approximately 13% of tobacco users in the state) [23]. The bias that older adults are unwilling or unable to engage in digital health platforms has contributed to their widespread exclusion in the development of digital treatments, including for cessation [26]. Consideration of older adults’ needs in the design and development of digital health interventions (eg, accommodating sensory and dexterity impairments) increases their treatment engagement [27]. Thus, to address this divide in the growing field of digital cessation treatment [16,22,23], detailed information is needed about how to best adapt these treatments for older adults.

This multimethods study aimed to collect detailed, hypothesis-generating information via semistructured qualitative interviews about the expectations and preferences for cessation digital treatment among older adults (aged 65+ years) who smoke cigarettes within an academic medical center. Interviews were supplemented with quantitative measures assessing sociodemographic characteristics, digital literacy, and physical health symptoms. Outcomes were intended to inform (1) the expected benefits and challenges of using digital cessation treatments across several modalities (ie, app-delivered, texting-based, or videoconferencing counseling) and (2) preferences and suggestions for digital treatments.

## Methods

### Study Sample and Recruitment

Participants were recruited by 1 of 2 methods. First, leveraging Medical University of South Carolina (MUSC) electronic health record (EHR) data, patients aged 65+ years currently smoking cigarettes were identified via a study recruitment report. Consistent with institutional review board (IRB) procedures, patients were contacted by phone and email. Those interested were asked to complete a phone-based screening questionnaire to determine eligibility. Second, patients identified as aged 65+ years were referred by providers from the MUSC Tobacco Treatment Program (ie, pharmacotherapy or behavioral counseling).

Eligibility criteria included being aged >65 years and having smoked 5+ cigarettes per day on more days than not in the past month. Individuals who recently quit (eg, within the past week) but had still smoked 5+ cigarettes per day on more days than not in the past month were included. Exclusion criteria included (1) non-English speaking, (2) no access to a telephone, (3) any self-reported cognitive impairment, and (4) having another household member enrolled in this study.

Following phone eligibility screening, participants completed electronic consent (eConsent) via a Health Insurance Portability and Accountability Act–compliant REDCap (Research Electronic Data Capture; Vanderbilt University) database [28]. Participants needed one-time access to the internet to read and sign the consent form electronically. Owning a personal device with internet access (ie, smartphone) was not required for participation. Study staff spoke with participants via telephone throughout the eConsent process, offering opportunities to ask questions about the consent form and instructions on how to access the form and sign electronically via a study link (if necessary). The remainder of the study procedures (ie, questionnaires and semistructured interviews) were administered via telephone.

### Procedures

Following informed consent, participants completed questionnaires and a semistructured interview (Multimedia Appendix 1).

### Semistructured Interviews

Following questionnaires, participants completed semistructured interviews with the lead researcher (MCF). These telephone interviews were approximately 45 minutes in length and audio recorded. Interviews consisted of a series of open-ended questions regarding cigarette smoking and quitting history, prior experiences with digital health treatment (eg, medical appointments or cessation treatment), and expectations and preferences for cessation treatment via different digital modalities (ie, app-delivered, texting-based, or videoconferencing counseling). Interviewees were also asked about expectations and preferences for digital treatments integrating social components (ie, social forums within an app-delivered treatment or group-based videoconferencing counseling).

### Measures

#### Demographics

Participants reported their age in years, gender, race, ethnicity, household income, education, marital status, and health insurance status.

#### Cigarette Use and Quitting History

Participants reported (yes or no) if they had recently quit cigarettes (defined as smoking 0 cigarettes, not even a puff, in the past 7 days), age of first cigarette, and use (yes or no) of other nicotine- and tobacco-containing products (ie, electronic cigarettes, smokeless tobacco, pipe, hookah, or cigars). Those who were currently smoking cigarettes reported the number of cigarettes they smoked per day and how soon after waking they had their first cigarette (within 5 minutes, 6-30 minutes, 30-60 minutes, and after 60 minutes). Participants also reported their motivation and confidence in quitting or remaining quit from cigarettes on a scale of 0-10, with 0 being the lowest motivation or confidence and 10 being the highest.

#### Physical Health

Participants reported whether any impairment or health problem limited any of their activities in any way (yes or no) using the Health-Related Quality of Life Activity Limitations Module [29]. If yes, participants reported the type of major limitation or health problem (arthritis or rheumatism, back or neck problems, fractures, or bone or joint injury, walking problem, lung or breathing problem, hearing problem, eye or vision problem, heart problem, stroke problem, hypertension or high blood pressure, diabetes, cancer, depression, anxiety, or emotional problem, and other impairment or problem). Finally, participants reported the presence of any sensory impairments (ie, visual or hearing; yes or no).

#### Digital Literacy

The Mobile Device Proficiency Questionnaire, a 16-item measure, assessed mobile device proficiency [30]. Scores range from 16 to 80, with higher scores indicating higher digital literacy.

#### Data Analysis

Recruitment was discontinued when data saturation was reached (no new themes were evident in 2 consecutive interviews) [31]. Audio-recorded interviews were deidentified by IRB-approved study staff and transcribed verbatim. An iterative approach to thematic analysis was used to code the qualitative interviews using NVivo software [32,33]. Further, 2 coders (MCF and RO) developed a codebook organized by interview prompts and the type of digital modality discussed. The same 2 coders then reviewed and coded a subsample of deidentified interviews (n=2) independently using the codebook. Using an iterative team-based approach, discrepancies were discussed, and the codebook was further refined. The codebook was updated (as needed) with each interview based on emerging themes. All deidentified interviews were independently double-coded (MCF, RO, and KP). Coders were reliable, with an agreement of 90%. An independent coder (MCF, RO, KP, or MRS) was chosen to resolve any coding discrepancies. Using NVivo software, codes facilitated the team’s (MCF, RO, KP, and MRS) identification, defining, and naming of themes meaningful to the expected benefits and challenges of digital cessation treatments, as well as preferences and considerations regarding digital design.

### Ethical Considerations

All study procedures were approved by the MUSC IRB (PRO00116590). Participants provided informed consent and were compensated US $50 via Amazon gift cards for participation. Interviews were audio recorded and transcribed. All data in this paper are deidentified.

## Results

### Sample Overview

In total, 20 participants completed semistructured interviews and Table 1 displays sociodemographic information. The majority (n=17, 85%) were currently smoking cigarettes and 3 (15%) participants had quit within the past month. Motivation to quit or remain quit was high (mean 7.9, SD 3.1), and half (n=10, 50%) reported having a physical limitation or impairment that limited their daily activities. Most identified as White (n=15, 75%), 20% (n=4) identified as Black or African American, and 5% (n=1) identified as Asian. All were non-Hispanic, and most were men (n=12, 60%) and not concurrently using other tobacco products (n=18, 90%). Household income was varied.

**Table 1:** Sample characteristics (N=20).

Characteristics			Value
**Age (years)**			
	Mean (SD)	70.7 (3)	
	Range	66-76	
**Gender, n (%)**			
	Men	12 (60)	
	Women	8 (40)	
**Race, n (%)**			
	White	15 (75)	
	Black or African American	4 (20)	
	Asian	1 (5)	
**Ethnicity, n (%)**			
	Non-Hispanic	20 (100)	
**Marital status, n (%)**			
	Married, partnered, or living as married	10 (50)	
	Not married	10 (50)	
**Household income (US $), n (%)**			
	<25,000	3 (15)	
	25,000-50,000	5 (25)	
	50,000-100,000	9 (45)	
	100,000-200,000	3 (15)	
**Smoking characteristics**			
	Recently quit, n (%)	3 (15)	
	Currently smoking, n (%)	17 (85)	
	Cigarettes per day, mean (SD)	10 (7.3)	
	Motivation to quit or remain quit, mean (SD)	7.9 (3.1)	
	Confidence to quit or remain quit, mean (SD)	6.2 (3.2)	
**Time to first cigarette (min), n (%)**			
	Within 5	1 (5)	
	6 to 30	7 (35)	
	31 to 60	5 (25)	
	After 60	4 (20)	
**Other tobacco co-use, n (%)**			
	Cigar	1 (5)	
	Electronic cigarette	1 (5)	
**Digital literacy, mean (SD)**			62.2 (20.7)
	Median	70.5	
	Range	16-80	
**Sensory impairments, n (%)**			
	Visual or hearing impairment	7 (35)	
**Physical limitation, n (%)**			
	Limited in everyday activities	10 (50)	
**Type of impairment or health problem, n (%)**			
	Back or neck problem	4 (20)	
	Lung or breathing problem	2 (10)	
	Cancer	2 (10)	
	Fractures, or bone or joint problem	1 (5)	
	Walking problem	1 (5)	

### Digital Cessation Treatments (All Modalities)

#### Overview

**Figure 1:**
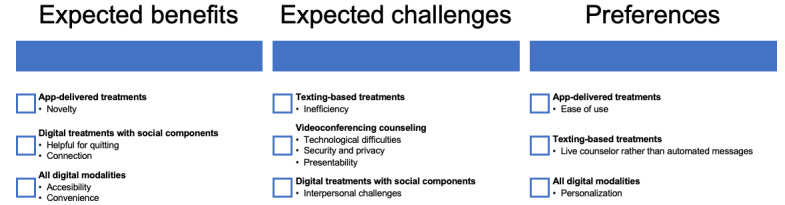
Qualitative themes regarding expectations and preferences for digital health modalities.

Thematic analysis identified 3 meaningful themes found across all digital modalities (app-delivered, texting-based, or videoconferencing counseling): convenience, accessibility, and personalization (Figure 1).

#### Accessibility

An expected benefit of digital platforms was easy access to treatment. Many participants did not have a car or were not able to drive independently and felt burdensome to friends and family who regularly provided transportation. Others found it inconvenient to arrange transportation (eg, public transit or car service) for themselves.

I don’t think I would come in person. That’s a lot of work if you want to see me once a week. It’s not like I can just get in the car and drive. I’d have to call transportation, call three days ahead, you know. Stuff like that. So, it’s a lot of work.

Aged 70 years, Black, woman, 3 cigarettes per day (reduced cigarettes per day in the past month), digital literacy 73/80

Because of these barriers, many discussed prioritizing in-person appointments only when necessary for medical procedures (eg, bloodwork). Thus, a benefit of behavioral treatment is that it does not require in-person services for physical procedures (such as bloodwork or urinalysis). In fact, some discussed interest in behavioral cessation support only if offered through digital platforms.

I prefer not to try to travel in areas that I don’t know. So, any place I go my husband basically drives me. I would not have done in-person only because that just puts more drain on my husband.

Aged 67 years, White, woman, 4 cigarettes per day, digital literacy 18/80

The physical act of traveling and sitting in waiting room chairs was painful for some individuals with physical impairments or chronic pain. Physical limitations (eg, breathing challenges or mobility impairments) were discussed as making in-person treatment uncomfortable and less preferred compared to digital health.

There’s the wait...the lost time [of in-person treatment]. And the chance of germs, not that I’m a germaphobe...and then the chairs are not really comfortable for long-term waiting...Sometimes I tell the receptionist I’m going out to sit in the car because my car seat is more comfortable than the chairs

Aged 70 years, White, woman, 28 cigarettes per day, digital literacy 32/80

#### Convenience

Another expected benefit of digital treatment was its convenience. Interviewees felt positive about the ability to access treatment when it was best for their schedule and avoiding in-person inconveniences (eg, parking). Participants appreciated the opportunity to wear comfortable clothes or be in the comfort of their homes when receiving cessation treatment. App-delivered and texting-based modalities were considered the most convenient (compared to videoconferencing) because they did not require scheduling an appointment with a provider. Participants liked that these modalities offered access to treatment 24/7.

I think an app would be more convenient than anything else. And you can use it when you needed.

Aged 73 years, White, man, 20 cigarettes per day, digital literacy 78/80

#### Personalization

Across all modalities, participants discussed a preference for treatment that was personalized to their individual experience with quitting. Concerns for app-delivered and text-based treatments were that these interventions might feel automated, impersonal, and not tailored to their unique quitting needs.

Automated is...it makes you feel, or you don’t feel like there’s a personal touch there.

Aged 70 years, White, woman, 7 cigarettes per day, digital literacy 79/80

Interviewees preferred individualized quit plans that could address their own craving triggers. Specifically for app-delivered interventions, suggestions included tailored educational materials for their age group and personalization features (eg, notifications during self-reported craving times or customized educational materials for their reasons for continued smoking).

If I get anxious, or sad over something, I’ll get a cigarette for my head to relax. So, I would want an app that would relate to those reasons.

Aged 70, White, woman, 7 cigarettes per day, digital literacy 79/80

For texting-based interventions, this sample preferred texting with a live counselor rather than receiving automated quitting tips. Participants believed automated messaging would likely be unhelpful given their long-term familiarity with standard advice to quit. Further, 1 participant who engaged with a texting-based cessation service discussed disliking the automated text messages.

The problem I’m having now is that I have a texting thing with [program name redacted] but it’s all automated. You know they’ll ask you a question and it’s just uh...a question that you should think of and if you try to respond to it, it just bounces back you know...it’s an automated system. Not personalized.

Aged 68 years, White, man, recently quit, digital literacy 59/80

Videoconference counseling was considered the most personalized form of treatment. Compared to telephone counseling, most preferred videoconferencing. Since many had had videoconferencing with their medical providers, they felt positive about seeing a provider’s facial expressions during the conversation. They believed videoconferencing facilitated a better relationship with their provider and thus would provide higher quality treatment (compared to telephone counseling).

I would rather video...where I can see someone on the computer on video. I just, it gives you, I guess you get more from the visual aspect of a person ...in my work, before I retired, I was [delivering] veterans counseling...so I depend upon visual cues as much as I do by voice.

Aged 69 years, Black, man, 5 cigarettes per day, digital literacy 79/80

### App-Delivered Interventions

#### Overview

Notably, 2 themes unique to app-delivered interventions were identified: novelty and ease of use (Figure 1). Only 1 participant had ever used an app-delivered cessation intervention; however, the majority (n=17, 85%) reported interest in using this type of treatment. Most (n=18, 90%) had used non–cessation-focused apps for a wide variety of reasons including social or communication, navigation, organization or calendar, games and entertainment, finances, shopping, and news. Only 2 interviewees were not currently using apps, both of whom had digital literacy scores <20 (out of 80) and did not own smartphones. However, both reported interest in this type of treatment.

#### Novelty

Many were unfamiliar with and curious about how apps could facilitate smoking cessation. Although this population used a variety of apps for other reasons (eg, entertainment, news, or navigation), fewer had used apps specifically for health reasons (eg, tracking steps). Participants were more familiar with videoconferencing and text messaging for cessation, and thus app-delivered treatments were the most unknown cessation modality. Aside from 1 participant who had used a smoking cessation app, no one had used an app-delivered treatment for any physical or mental health problem. Yet, interviewees were open to trying this novel treatment and evaluating its usefulness.

I think that’s interesting...um I mean I’d be curious about it. That doesn’t mean I’d be ongoing using an app...but I’d be curious if that makes sense. If I find it to be helpful, I’d find that very interesting for me to figure out.

Aged 76 years, White, woman, 10 cigarettes per day, digital literacy 71/80

Participants commonly described frustration with their inability to quit cigarettes, and an eagerness to try any means for quitting. An expected benefit of app-delivered treatments was that they offered a novel strategy for treatment.

An app was most appealing if offering novel resources beyond standard advice to quit. Most participants described long-term familiarity with standard education about the harms of smoking. Individuals were more open to trying strategies or educational materials beyond this standard content.

If it’s going to be the same tips that you get “uh smoking is bad for your health, smoking causes cancer”, et cetera, et cetera. I would want it to be something other than the routine that I’ve seen for the last 40 years

Aged 70 years, White, woman, 7 cigarettes per day, digital literacy 79/80

#### Ease of Use

Participants were concerned that an app-delivered treatment might be overly complicated and challenging to navigate. Interviewees referenced frustrating experiences with apps that contained too much content, did not function properly, or were difficult to understand. Some discussed enlarging font size on apps that they used for noncessation reasons. Many said they would not have the patience to engage with an app that had an overwhelming amount of content, tabs, or buttons to click.

Well, if it’s a complicated app, most of us that are 75 years old are going to have difficulty maneuvering through it. But if it’s something real simple, like going [to] the next page, next page, or click on this to learn this, you know...uh depends on how you maneuver through the app.

Aged 75 years, White, woman, 18 cigarettes per day, digital literacy 47/80

### Texting-Based Interventions

#### Overview

Notably, 1 unique theme emerged for text-based interventions: inefficiency (Figure 1). All participants had at least some experience with texting before this study; however, comfort with and interest in texting ranged widely. Only 1 participant had used a texting-based cessation intervention before this study (a different participant than the one who used an app-delivered treatment). Half (n=10, 50%) said they would be interested in this type of treatment.

#### Inefficiency

A negative expectation of texting-based interventions was that the action of sending text messages would be inefficient for communication and treatment engagement. Many believed that they texted more slowly and with more difficulty than the average person, thus making texting frustrating. Texting was discussed as necessary for brief communication (eg, scheduling); however, sending text messages was not an enjoyable activity. Texting was considered burdensome and an unappealing option for treatment engagement.

I try to stay as far away from texting as I can. I’m a hunt and peck typer...I don’t text a lot...I guess I can do it when I have to. But I’m not that big for it. I try not to text. I’ll call you first.

Aged 69 years, Black, man, 5 cigarettes per day, digital literacy 79/80

### Videoconferencing With Cessation Counselor

#### Overview

Notably, 3 unique themes for videoconferencing counseling were identified: presentability, security and privacy, and technological difficulties (Figure 1). Compared to other digital modalities, participants were most familiar with videoconferencing. More than half (n=12, 60%) had a history of videoconferencing with their medical providers. Many discussed using videoconferencing platforms for work-related or social activities. Only 1 participant had used videoconferencing for cessation counseling (a different individual from the 2 participants who had used an app-delivered and texting-based cessation treatment). Most (n=17, 85%) said they would be interested in this type of treatment.

#### Presentability

When videoconferencing (rather than telephone counseling or another digital modality), a negative expectation was the time and effort for on-screen presentability (eg, getting dressed or applying makeup). Although participants felt that visibly seeing their counselor would improve the quality of their cessation care, presentability was an expected barrier (depending on their time and level of motivation for the day). Interviewees also discussed feeling self-conscious when showing their face on videoconferencing screens. For app-delivered or texting-based treatments, interviewees appreciated not needing to shower or change clothes to engage with content.

As far as the Skype-in’ and ...I don’t like to do it because I’m very self-conscious of all my wrinkles. Um, and I look horrible on those screens.

Aged 75 years, White, woman, 18 cigarettes per day, digital literacy 47/80

#### Privacy and Security

A concern was that personal information shared in videoconferencing sessions could be overheard (in either the patient or provider’s environment) and that counselor notes might be shared. Participants said they wanted to know where counseling notes were stored and if they would be shared with other providers. Interviewees appreciated that app-delivered or texting-based treatments might not require them to disclose personal information (eg, last name or address) and were more anonymous than videoconferencing.

Security. You just hear about it all the time...using technology I’m just kind of leery of it...personal information getting out. Well, they ask about your personal information, social security questions etcetera, etcetera, I would worry about this immensely.

Aged 73 years, White, man, 20 cigarettes per day, digital literacy 78/80

#### Technological Difficulties

An expected challenge was navigating technology in the setup or duration of a videoconferencing appointment. Interviewees believed technology-based difficulties would be less common with app-delivered or text-based modalities. Many had prior challenges setting up videoconferencing sessions with their medical providers or had internet connection issues during the visit. Technology-based issues were frustrating and negatively impacted the quality of care and overall experience.

I was trying to log into the network, and the doctor’s office, they had um a link and I kept trying to get it. But evidently either I was doing something wrong, or whatever it just never worked so I kind of gave up on that...you know, maybe it was just new to me, or new to them. It just didn’t work.

Aged 69 years, Black, man, 5 cigarettes per day, digital literacy 79/80

### Digital Health Treatments With Social Components

#### Overview

Notably, 3 themes emerged regarding digital cessation treatments that integrated social components: helpful for quitting, connection, and interpersonal challenges (Figure 1). No participant reported prior experience with digital cessation treatments that integrated social components.

#### Helpful for Quitting

Some interviewees had personal experiences with in-person group counseling for other mental or physical health problems. Others had friends or family who had benefited from group counseling. Referencing these experiences, interviewees believed that listening to others quitting might be helpful for their own quit success. For app-delivered treatments, participants commonly discussed that the main benefit of a social forum would be to learn new strategies and tips for trying to quit.

Never thought about that, but that might be helpful...to hear other people...the ones that are dealing with the problem...and their tried solutions...and failed solutions and why they think it worked and why they didn’t think it worked and how that’d compare to my life you know

Aged 70 years, White, woman, 28 cigarettes per day, digital literacy 32/80

#### Connection

An expected benefit was the ability to build connections with others in cessation treatment. For group videoconferencing (rather than app-delivered treatments with social forums), interviewees appreciated the idea of meeting others with the same goals. Participants mentioned that others might provide accountability and encouragement during their quit attempts.

I know it’s essential...to stop smoking its essential to network...okay and I know that because of AA and NA and the way all those 12-step programs work...there’s [only] one part of it [that] is the knowledge, if you will, and then it’s the fellowship, the interacting with other people trying to quit...it has an equally if not more value in um...of me facing myself...let me see if I can explain it...that I call someone before I smoke that cigarette that holds me accountable and if I reach that level of accountability with cigarettes then I might have a chance of putting em’ down and I don’t think there’s any replacement for that personal interaction between two people trying to accomplish the same thing

Aged 69 years, White, man, recently reducing cigarette use (down to 1 cigarette per day), digital literacy 69/80

#### Interpersonal Challenges

Interviewees were concerned about potential interpersonal challenges. They felt negatively about not having a chance to speak (in group videoconferencing) if others were too talkative. Regarding social forums in apps, participants did not want their personal information shared in the case that another patient might contact them outside of the platform. Interviewees were also concerned that the anonymity of app-delivered social forums (vs group videoconferencing) would allow for negative comments and arguments.

I would not participate. And I mean...things unfortunately on social media turn ugly so quick it’s unbelievable. Now, I do not post anything or...well rarely do I post anything on my [social media] page. I do have a business [social media] page that I post on and I run social media advertising off of. But I mean, the ugliness of social media just blows my mind...so that’s just a real turn off for me...Because they’re not sitting there next to the person. It’s easy to sit behind a keyboard and blast somebody than to be sitting next to them

Aged 68 years, White, man, recently quit, digital literacy 80/80

## Discussion

### Principal Findings

This multimethods study explored the expectations and preferences for digital tobacco treatment among a population of older adults currently smoking or who had recently quit cigarettes. Only 3 participants had engaged with a digital tobacco cessation treatment before this study, with most unaware of these types of treatments. Technology use for non–cessation-related reasons was common (ie, texting, apps, or videoconferencing with medical providers). Digital literacy varied widely, yet the majority had relatively high scores (median 70.5, range 16-80 out of 80). Most (n=17, 85%) were interested in either an app-delivered treatment or videoconferencing counseling, and half (n=10, 50%) would try a texting-based treatment. Even individuals with lower digital literacy and who did not own smartphones were interested in digital cessation treatments. These results challenge the bias that older adults are uninterested in digital health treatments [26]. Thematic analysis identified 3 meaningful themes across all digital modalities (app-delivered, text-based, or videoconferencing counseling): convenience, accessibility, and personalization. Compared to in-person treatment, digital treatment was regarded as more accessible to those with physical impairments and without transportation. This sample felt positively about accessing treatment from their homes and avoiding in-person inconveniences (eg, parking). Yet, digital health treatments that relied on automated messaging, used a “one size fits all” approach to treatment, or delivered only standard advice to quit were unappealing. Personalized content was consistently preferred for digital treatment. This preference is consistent with other age groups, in which tailored content (eg, scheduling personal quit date) in app-delivered cessation treatment is related to app popularity and user-rated quality [34].

App-delivered treatments for cessation were appealing by offering a novel and unfamiliar behavioral strategy for quitting. This sample commonly had a long history of quit attempts and was overly familiar with the harms of smoking. Perhaps desirable for this age group, apps can provide novel and personalized strategies (eg, triggers or reasons for quitting) and content (eg, quitting in older age) beyond standard education [16]. Other ways to personalize content to experiences more common among this age group might include acknowledgment of long histories of quit attempts, low self-efficacy, negative experiences with cessation pharmacotherapy, and fatigue with standard advice. Consistent with the literature detailing aging-related considerations for digital health [35], app design suggestions included simplicity, easy navigation, and enlarged font size. This sample preferred less texting-based engagement within apps, which might be indicative of dexterity barriers for digital health treatment among this age group (eg, aging-related motor decline or stiffer joints) [35]. Thus, previous challenges with using apps were memorable and informed their preferences for future treatment. Despite usability concerns, participants were open and curious about using this modality for cessation. These findings are consistent with a pilot trial, in which older adults found a standard (not tailored for the unique needs of older adults) cessation app to be moderately acceptable (median 3 out of 5 stars) [36]. Potential ways to further increase the acceptability of app-delivered treatments include prioritizing personalized content and design ease of use.

Texting-based interventions were the least preferred digital modality, perhaps given disinterest in the action of sending text messages. All participants had texted before this study but many found it to be inefficient for communication and unenjoyable. Participants were also uninterested in interventions that relied on automated messaging and content. These findings are inconsistent with a prior feasibility trial, in which 57% of older adults (aged 60+ years) found a text-based cessation intervention useful [37]. Notably, this text-based intervention was tailored to the individual’s harm reduction progress and did not heavily rely on participant texting for engagement [37]. However, these results suggest that older adults might initially prefer other digital modalities for cessation.

Expectations for videoconferencing counseling were largely informed by past experiences videoconferencing with medical providers. Beyond the general appeal of digital health platforms, participants believed this treatment modality would provide the most personalized content. Expected challenges included navigating technological difficulties, lack of privacy or security, and appearing presentable onscreen. Despite these concerns, videoconferencing was preferred over telephone counseling given its potential to provide better quality of care. Although the literature is limited on this topic [38], recent evidence suggests that cessation videoconferencing might be more effective than telephone counseling for increasing short-term quit success [39]. Cessation counselors should consider offering videoconferencing for older patients, which might be a more appealing form of treatment for this age group.

No participant had experience with digital cessation treatments incorporating social components (group-based counseling or social forums). Expected benefits included usefulness for quitting (eg, encouragement and accountability or new tips for quitting) and social connection. Yet, participants were concerned about possible interpersonal challenges (eg, negativity in social forums). Digital social forums might benefit from moderated or asynchronous messaging for this age group. Many had positive experiences with addiction treatment (eg, Alcoholics Anonymous) and were thus open to group-based videoconferencing for cessation. Overall, because this population discussed disinterest with standard advice to quit, cessation treatment integrating social components might be novel and appealing to this age group.

Beyond disinterest in standard education and nontailored automated content, participants did not discuss specific cessation treatment components or topics that they would prefer within digital interventions. Participants might have been unable to identify preferences for more detailed content for a few reasons. First, the purpose of this study was not to elicit feedback on any existing cessation program, but rather to gain hypothesis-generating information about expectations and preferences for digital cessation interventions broadly. Thus, participants were not given any descriptions of existing treatments for which to provide feedback. Further, most of this sample had limited prior experiences with digital cessation programs and might have had difficulty envisioning these types of treatments. When developing digital cessation treatments, it will be important for future researchers to elicit more detailed and specific feedback from this population to guide the refinement process.

Results should be interpreted with study limitations in mind. This sample was recruited from an academic medical center given that older adults have much higher rates of health care use than their younger counterparts [40] and thus medical settings can play an important role in reaching older adults who smoke cigarettes. However, findings might not be generalizable to other geographic locations or populations without access to medical services (n=20, 100% of this population had health insurance). Although digital literacy varied, the majority had relatively high scores. Further, this study required 1-time access to the internet for eConsent. Although 2 participants borrowed mobile devices from family members for eConsent (as owning a smartphone or regular access to the internet was not required for participation), results might be most representative of older adults with access to technology and greater digital literacy. Even though men have higher smoking rates than women [7], our sample included 60% (n=12) men and thus might be less generalizable to women. Additionally, this study was not inclusive of all types of digital modalities. Future studies should elicit feedback regarding other digital treatments (eg, remote carbon monoxide sensors), particularly as this field continues to grow and adapt to new technologies. Finally, it will be important to ascertain the best means by which to refer older adults to digital cessation programs, as well as the preferred devices (eg, tablets as compared to desktop computers). For example, older adults might be even more likely to engage in digital programs if their medical providers refer them to these treatments. Future studies should qualitatively and systematically evaluate preferences for digital health treatments among this heterogeneous age group within large diverse samples (by gender, race, ethnicity, or socioeconomic status) and might consider eliciting feedback on existing freely available digital cessation programs.

### Conclusions

Older adults are the most impacted by tobacco morbidity and mortality and, therefore, should be prioritized in the growing field of digital cessation treatment. Given a long history of quit attempts and familiarity with standard advice to quit, digital health treatments might offer appealing new behavioral approaches to quitting for this age group. This sample was interested and willing to engage with digital platforms, expecting them to be more accessible and convenient than in-person treatment. Preferences included simple and easy-to-navigate digital designs and personalized rather than automated content. Findings challenge the bias that older adults are uninterested or unwilling to engage with digital treatments for behavioral health. Clinicians and researchers should prioritize the inclusivity of older adults in the development and dissemination of digital cessation treatments.

## Appendix

Multimedia Appendix 1 Semistructured interview guide.

## Abbreviations

eConsent: electronic consent
IRB: Institutional Review Board
MUSC: Medical University of South Carolina
NA: Narcotics Anonymous
REDCap: Research Electronic Data Capture
