# Supplementary material for: Expectations and Preferences for Digital Cessation Treatment: Multimethods Study Among Older Adults Who Smoke Cigarettes
Source: J Med Internet Res. 2024 Aug 28;26:e52919. doi: 10.2196/52919 (PMC11391153; doi:10.2196/52919)
Supplement: Multimedia Appendix 1 [file jmir_v26i1e52919_app1.docx]

**Qualitative Interview (60 minutes)**

Cigarette Use & Quitting

1. How interested in quitting cigarettes are you?

A. (*If yes*) Why are you interested in quitting?

3. Have you ever tried to quit before?

A. (*If yes*) How was that experience for you?

5. Have you ever been given advice by your doctor to quit?

A. (*If yes*) What was that advice like?

Medical and Mental Health Telehealth Visit

*Now I’m going to ask about your experiences with telehealth. Telehealth means communicating health-related information with your doctors or other healthcare professionals through technology (video, computer, mobile devices).*

1. Have you ever used telehealth for an appointment with a medical or mental health doctor/provider?

2. (*If yes*)

A. How was this experience for you?

B. What did you like about this experience?

C. What did you dislike about this experience?

D. How did this/these visits compare to an in-person appointment?

3. (*If no*)

A. Why not?

B. Would you be interested in this type of medical visit?

Tobacco Cessation Treatment Preferences

1. (*If interested in quitting cigarettes)*

A. How interested in receiving counseling for quitting are you?

B. How interested in counseling for quitting smoking via telehealth are you?

2. (*If not interested in quitting cigarettes*)

A. If you were to receive counseling for quitting smoking, how interested in counseling via telehealth would you be?

3. If you were to receive counseling for quitting smoking, what type of telehealth would you prefer (video, telephone, text messaging, smartphone applications)?

4. Why would you prefer this type of telehealth?

5. For a telephone session, one-on-one with a counselor,

A. What would be the positives?

B. What would be the negatives?

C. How would this compare to in-person?

6.. For text messaging, one-on-one with a counselor,

A. What would be the positives?

B. What would be the negatives?

C. How would this compare to in-person?

7. For an individual video session one-on-one with a counselor,

A. What would be the positives?

B. What would be the negatives?

C. Do you have any concerns about this type of visit?

C. How would this compare to in-person?

D. How could a counselor make the video session better/more convenient for you?

E. What are special considerations cessation counselors should consider when conducting video session with someone over the age of 65?

F. What would you think about a meeting, prior to your session with a counselor, to help you get set-up with the technology for the visit?

a. What would be the positives of this?

b. What would be the negatives of this?

8. For a group video session with other smokers over the age of 65 and a counselor,

A. What would be the positives?

B. What would be the negatives?

C. Do you have any concerns about this type of visit?

D. How would this compare to an in-person group session?

8. For smartphone applications that helps you quit smoking,

A. What would be the positives?

B. What would be the negatives?

C. How would this compare to an in-person visit with a counselor?

D. What type of features would you like in an app?

E. How would you feel about video content on an app?

F. How would you feel about an app that helps you track your goals?

G. How would you feel about an app that connects you to other smokers?

H. How would you feel about an app that helps you track the number of cigarettes you smoke each day?

I. How would you feel about a smartphone application that also helps you manage physical pain?

J. How would you feel about a smartphone application that also helps you manage all your prescription medications?

K. How would you feel about a smartphone application that has larger font size to improve readability?
